# Editorial: Transcatheter mitral and tricuspid valve therapies

**DOI:** 10.3389/fcvm.2023.1329387

**Published:** 2023-12-04

**Authors:** Omar Chehab, Ronak Rajani, Simon Redwood, Bernard Prendergast, Tiffany Patterson

**Affiliations:** Department of Cardiovascular Medicine, St Thomas’ Hospital, London, United Kingdom

**Keywords:** structural heart intervention, mitral regurgitation, tricuspid regurgitation, aortic stenosis, transcatheter aorta valve replacement, transcatheter edge to edge repair, transcatheter valve replacement/implantation, valvular heart disease

**Editorial on the Research Topic** Transcatheter mitral and tricuspid valve therapies

Transcatheter valve therapies have emerged as a viable treatments option for patients deemed high risk for conventional surgery. Whilst transcatheter aortic valve implantation (TAVI) is now established as the standard of care in high and intermediate risk aortic stenosis patients (1, 2), the mitral and tricuspid frontiers have proved to be more challenging. Anatomical heterogeneity, device development, refining patient selection and until recently the absence of randomised data have all been contributing factors. For mitral regurgitation transcatheter edge to edge repair (TEER) now benefits from positive randomised control trial (RCT) data (3) along with significant advancements in device technology. Transcatheter mitral valve replacement (TMVR) options are also making steady progress. More recently, having previously been referred to as the “forgotten valve”, the vast unmet clinical need associated with tricuspid regurgitation has become the central focus of the structural heart community. There were positive signals of benefit in the only RCT of TEER (4) and tricuspid transcatheter valve replacement (TTVR) has shown great promise so far with randomised data coming soon (5). This research topic aims to explore a broad range of areas within mitral and tricuspid intervention with a particular focus on optimising outcomes, identification of favourable responders and challenging patient cohorts.

In degenerative mitral regurgitation (DMR), a significant proportion of patients do not derive symptomatic benefit or reverse remodel following TEER. Selecting the right patient for the right intervention to maximise benefit and potential for a durable result is vital. Central to procedural success and minimising residual regurgitation is detailed prior anatomical assessment and skilled interventional imaging using transoesophageal echocardiography (TOE). In their mini review, Hirasawa and Izumo outline the role of three-dimensional (3D) TOE in assessing mitral valve (MV) geometry and dimensions with the use multiplanar reconstruction. Intraprocedural 3D TOE imaging also aids in the improved visualisation and quantification of residual MR jets following device deployment.

MR is one of the commonest abnormalities associated with rheumatic heart disease (RHD) along with mitral stenosis. Gomes et al. looked at predictors of MR severity progression In their study of 539 patients with RHD. They found that age and LA volume were the most predictive factors with patients exhibiting features of both degenerative and functional MR (FMR). Mitral annular calcification (MAC) associated with MV dysfunction also continues to pose a significant challenge to cardiologists and surgeons alike. The patients tend to more comorbid, and both percutaneous and surgical treatments are not without significant procedural risk. Where possible hybrid procedures that involve minimal decalcification or transcatheter approaches are used although patients continue to have poor longer-term outcomes (6). In their mini review, Ascione and Denti et al. outline the growing role of transcatheter mitral valve replacement with the Tendyne™ valve (Abbott, MN, United States), along with the various approaches to mitigating the risk of left ventricular outflow obstruction.

Great progress has been made in the management of secondary MR with the COAPT study paving the way for TEER in carefully selected patients. Despite this, these patients still have a poor prognosis and a significant proportion do not derive benefit from MR reduction. Further emphasising the need to better understand the disease itself and how we go about patient selection (7). In their systematic review and meta-regression analysis Shi et al. sought to assess clinical predictors following percutaneous MV repair using both annuloplasty and TEER in patients with FMR. Their findings once again linking LV volumes to mortality emphasise the importance of intervening early before excessive eccentric remodelling occurs. Greater focus has been placed on the right ventricle given its prognostic importance more broadly. The review article by Stolz et al. highlights the challenges of RV assessment using 2D echocardiography, and the added value of RV to pulmonary artery coupling in predicting outcomes in both mitral and tricuspid TEER patients. Finally, acute MR is a new frontier for mitral TEER. Papillary muscle rupture in the context of myocardial infarction is potentially a life-threatening complication and the management of such patients is very challenging. Both medical therapy and emergent surgery are associated with high rates of morbidity and mortality, paving the way for TEER as a less invasive alternative. Estevez-Loureiro et al. in their review of the topic outline the current role of transcatheter interventions in this setting with a summary of the latest registry data.

Finally, moving onto the previously neglected area of tricuspid regurgitation, we have seen a major effort by the structural heart community to focus attention on this vastly undertreated patient population. Recently in the first and only RCT, TV TEER demonstrated an improvement in quality of life in line with the degree of TR reduction (4). Tricuspid patients tend to have multiple comorbidities, advanced left heart disease or both, making them less than ideal surgical candidates. Fortunately, TTVR is showing promise with highly efficacious TR reduction and fewer anatomical obstacles compared to TMVR. That said, there is some hesitation regarding the RV's susceptibility to the abrupt surge in RV afterload that may follow TR elimination. Sala et al. discuss this in detail in their review highlighting the inadequacy of longitudinal 2D markers of contractility when compared to 3D volumetric RV assessment. They also emphasise the value to assessing RV ejection fraction, and its relationship to the pulmonary circulation as potential discriminators of patients that may suffer from afterload mismatch and RV failure.

Mitral and tricuspid valve disease are increasingly common as society ages and are directly associated with significant morbidity and mortality. Guideline directed medical therapy has an important role in some instances and limited in others (8), leaving transcatheter therapies with an important part to play. This research topic seeks to explore the important role of advanced imaging in refining procedural outcomes and patient selection. It also reviews the management of more challenging patient populations and new indications for TEER. Finally, the right heart is now firmly in focus as an important indicator of prognosis and tricuspid therapies rapidly coming to the aid of a previously underserved patient population.
